# Pulsed-field ablation for non-pulmonary vein foci of immediate recurrence of atrial fibrillation originating from the left arial inferior wall directly adjacent to the esophagus

**DOI:** 10.1016/j.hrcr.2025.04.009

**Published:** 2025-04-17

**Authors:** Hirofumi Arai, Yuichiro Sagawa, Atsuhito Oda, Kazuya Murata, Tetsuo Sasano, Yasuteru Yamauchi

**Affiliations:** 1 Department of Cardiology, Japan Red Cross Yokohama City Bay Hospital, Shinyamashita, Yokohama, Kanagawa, Japan; 2 Department of Cardiovascular Medicine, Institute of Science Tokyo, Yushima, Bunkyo, Tokyo, Japan

**Keywords:** Pulsed-field ablation, Atrial fibrillation, Immediate recurrence of atrial fibrillation, Nonpulmonary vein foci, Esophageal injury

**Key Teaching Points**

• Catheter ablation targeting non-pulmonary vein (PV) foci of atrial fibrillation, particularly those originating from the left atrial inferior wall directly adjacent to the esophagus, presents significant challenges because of the risk of esophageal injury.
• Pulsed-field ablation (PFA) is a promising option for minimizing the risk of esophageal injury in non-PV foci.
• PFA can safely eliminate non-PV foci in the left atrial inferior wall.

## Introduction

Pulsed-field ablation (PFA) introduces electroporation using a high-voltage electrical field as a nonthermal energy source. Although electroporation can be reversed, prolonged exposure to a high-voltage electrical field induces irreversible electroporation, resulting in cell death. PFA demonstrates myocardial tissue selectivity, thereby reducing the risk of esophageal injury compared with thermal ablations such as radiofrequency (RF) catheter ablation and cryoballoon ablation. The efficacy of PFA for pulmonary vein isolation (PVI) has been reported; however, its utility for nonpulmonary vein (PV) foci in atrial fibrillation (AF) remains less established. This case report highlights the use of PFA to target non-PV foci originating from the left atrial inferior wall, which is directly adjacent to the esophagus.

## Case report

A 67-year-old man with a history of catheter ablation for persistent AF, including PVI, left atrial posterior wall isolation with roof line cryoballoon ablation, floor line RF ablation, and cavotricuspid isthmus ablation, experienced recurrent paroxysmal AF (PAF). Repeat catheter ablation for PAF recurrence was performed under total intravenous anesthesia with propofol. The i-gel supraglottic airway device (Intersurgical, Wokingham, Berkshire, UK) was used for mechanical ventilation, and Esophastar (Japan Lifeline, Tokyo, Japan), an esophageal luminal temperature catheter, was inserted via i-gel through the esophagus. Three-dimensional (3D) electro-anatomical mapping (EAM) was conducted using an Advisor HD Grid mapping catheter (Abbott, Chicago, IL).

**Figure 1:**
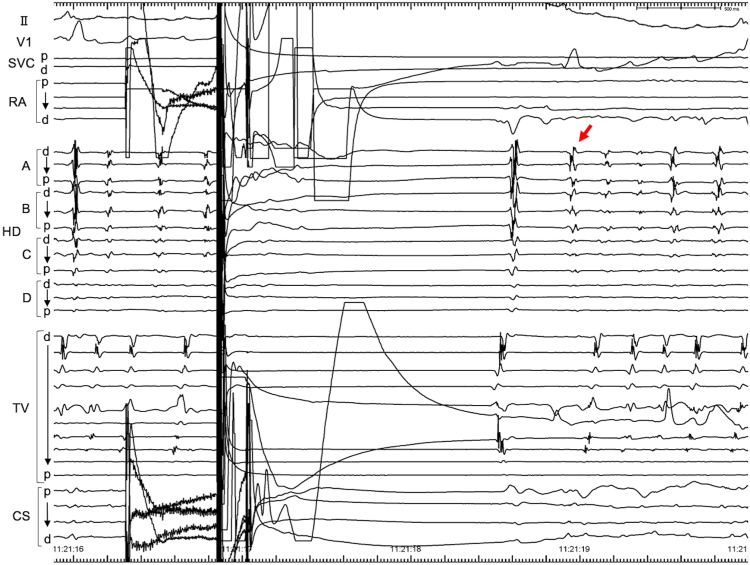
Immediate recurrence of atrial fibrillation (AF). AF was spontaneously induced during 3-dimensional (3D) electro-anatomic mapping. Intracardiac defibrillation terminated the AF; however, it recurred immediately after. *Red arrow* indicates the initiation of AF. CS = coronary sinus; d = distal; HD = HD grid mapping catheter; p = proximal; RA = right atrium; SVC = superior vena cava; TV = tricuspid valve.

**Figure 2:**
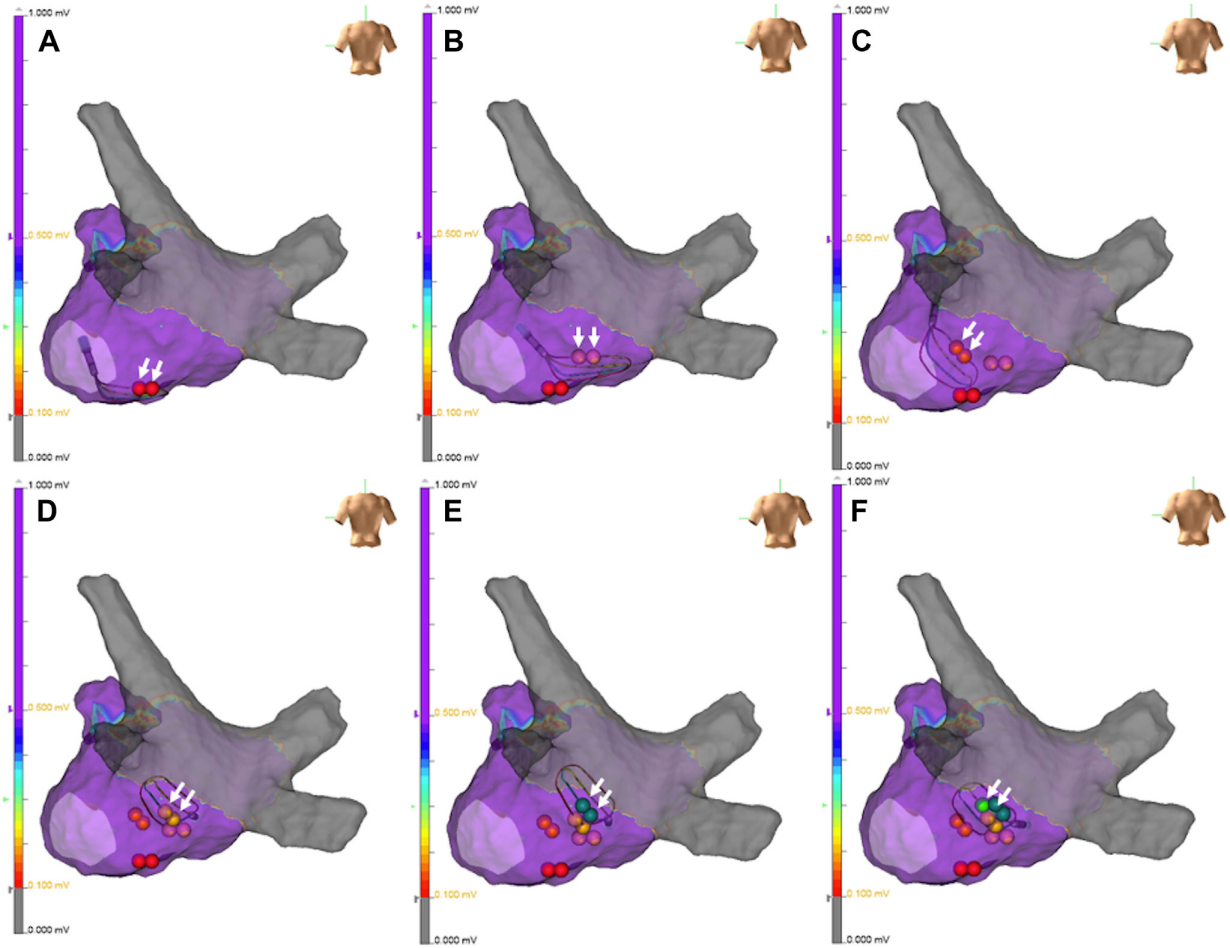
Self-reference mapping of the nonpulmonary vein foci. Self-reference mapping was performed to identify the location of non–pulmonary vein (PV) foci. The earliest activation site (EAS) at the initiation of atrial fibrillation (AF) was identified using an HD Grid mapping catheter. The new tags in each panel indicated by *white arrows* were the EAS in the HD Grid mapping catheter after each cardioversion. The catheter was gradually advanced toward the EAS from panels **A** to **F**, ultimately pinpointing the origin of the non-PV foci in the inferior wall of the left atrium.

**Figure 3:**
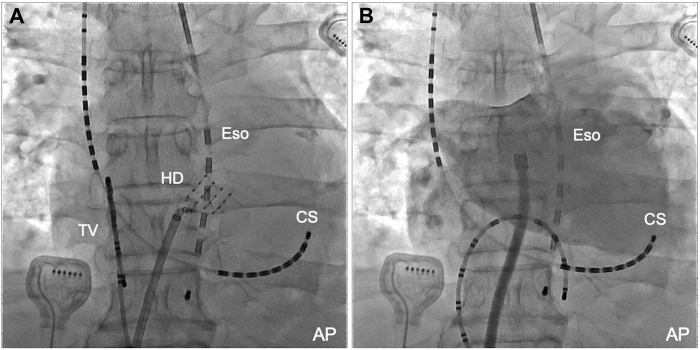
Origin of the non-pulmonary vein (PV) foci and the contrast imaging of the left atrium. The HD Grid mapping catheter was positioned at the origin of the non-PV foci, located directly adjacent to the esophagus (**A**). Contrast imaging of the left atrium provided detailed anatomic information (**B**). AP = anteroposterior view; CS = coronary sinus; Eso = esophageal luminal temperature catheter; HD = HD Grid mapping catheter; TV = tricuspid valve.

3D EAM confirmed no recurrence of PVI, posterior wall isolation, or cavotricuspid isthmus conduction; however, AF was spontaneously initiated during left atrial mapping. Repeated intracardiac electrical cardioversion with a 10 J shock using the BeeAT catheter (Japan Lifeline) through the coronary sinus, and the Shock AT system (Japan Lifeline) successfully terminated AF each time; however, AF recurred almost immediately after each attempt, resulting in the immediate recurrence of AF, in which sinus rhythm could not be maintained (Figure 1). Non-PV foci were suspected. The origin of the non-PV foci was confirmed by self-reference mapping with the Advisor HD Grid mapping catheter (Figure 2) and was localized to the left atrial inferior wall adjacent to the esophageal luminal temperature catheter (Figure 3). PFA was selected for treatment because of safety considerations, specifically because of the potential risk of esophageal injury associated with RF ablation.

**Figure 4:**
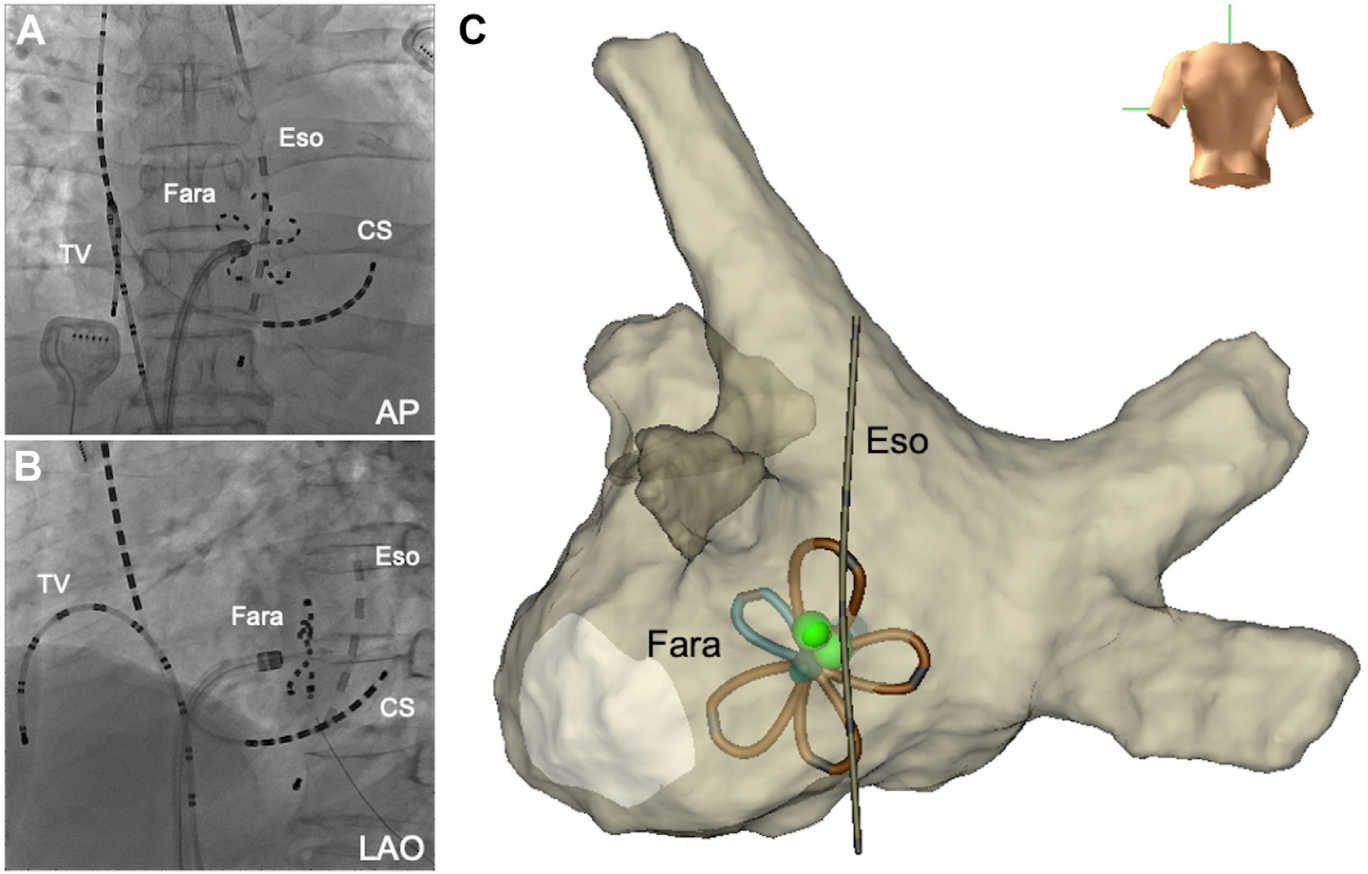
Pulsed-field ablation for the non-pulmonary vein (PV) foci. Pulsed-field ablation (PFA) was performed for non-PV foci located directly adjacent to the esophagus. The position of the Farawave catheter in its flower configuration was confirmed using fluoroscopy (**A, B**) and 3-dimensional (3D) electroanatomic mapping (EAM) (**C**). The green tags on the 3D EAM indicate the origin of the non-PV foci. The Farawave was positioned adjacent to the esophageal luminal temperature catheter. AP = anteroposterior view; CS = coronary sinus; Eso = esophageal luminal temperature catheter; Fara = Farawave; LAO = left anterior oblique view; TV = tricuspid valve.

**Figure 5:**
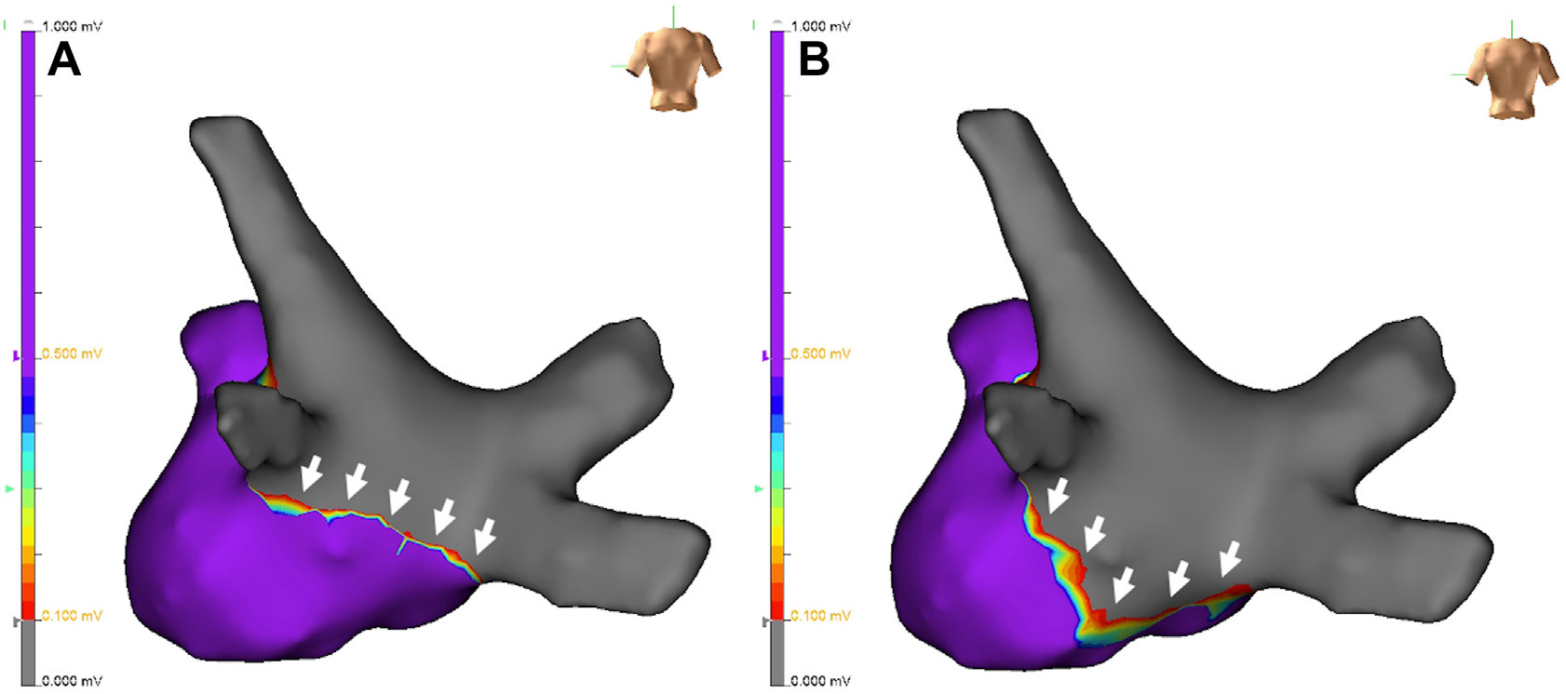
Three-dimensional electro-anatomic mapping before and after the pulsed-field ablation. Three-dimensional electro-anatomic mapping before (**A**) and after (**B**) pulsed-field ablation confirmed the successful ablation of non-pulmonary vein foci. The floor line (*white arrows*) was extended to the inferior wall of the left atrium.

The Farawave ablation catheter (Boston Scientific, Marlborough, MA) was advanced through the Faradrive (Boston Scientific). PFA with a voltage of 2.0 kV was applied to the non-PV foci using the Farapulse PFA system (Boston Scientific) in the flower configuration of the Farawave catheter. Two initial applications were performed, followed by 2 additional applications after catheter rotation (Figure 4). No obvious esophageal temperature rise was observed during the applications. Subsequent intracardiac electrical cardioversion successfully terminated AF, and sinus rhythm was maintained. Immediate recurrence of AF was resolved using PFA to target non-PV foci. Each of the 2 additional PFA applications was performed on the right, left, and inferior sides of the origin of the non-PV foci. Postprocedure 3D EAM confirmed successful ablation of non-PV foci originating from the left atrial inferior wall directly adjacent to the esophagus and isolation of the inferior wall region approximately 1 cm below the floor line (Figure 5). The patient was discharged without symptoms suggestive of esophageal injury, and no AF recurrence was observed. The patient had no AF recurrence during a 4-month follow-up after the procedure.

## Discussion

PFA is a novel ablation system using a nonthermal energy source, contrasting with conventional thermal ablation methods such as RF and cryoballoon ablation. Although most reports on PFA have focused on PVI, its application for non-PV foci remains unexplored. This case represents the first reported use of PFA for non-PV foci originating from the left atrial inferior wall, directly adjacent to the esophagus.

The Farapulse (Boston Scientific, Menlo Park, CA) PFA system employs a pentaspline catheter (Farawave) adjustable to either a basket or flower configuration. The efficacy of the Farapulse system for PAF has been reported,^1^ showing outcomes comparable to those of conventional thermal ablation.^2^ Although PFA for non-PV foci in the left atrial inferior wall has not been previously reported, its use for left atrial posterior wall ablation with the Farapulse system in the flower configuration has been described.^3^, ^4^, ^5^, ^6^, ^7^, ^8^ This method was applied in the current case to ablate non-PV foci located at the left atrial inferior wall below the floor line of the left atrial posterior wall, achieving successful elimination of non-PV foci.

PFA demonstrates myocardial tissue selectivity and can be performed without collateral damage to the surrounding tissues such as the esophagus and phrenic nerve.^9^ Animal studies have shown no histopathological changes in the esophagus of swine models after PFA,^10^ and clinical trials have reported no esophageal injuries.^11^ PFA offers a safer alternative for RF ablation for lesions adjacent to the esophagus because of the low risk of esophageal injury.

Additionally, PFA penetrates existing RF lesions and create deep lesions beneath prior RF lesions, unlike RF ablation, which has limited effectiveness in such scenarios.^12^ In this case, the origin of non-PV foci beneath a prior RF floor line necessitated frequent RF applications for effective lesion formation, thereby significantly increasing the risk of esophageal injury. Consequently, a treatment method minimizing esophageal injury risk was required. PFA can create an effective lesion even in the presence of prior RF lesion with low risk of collateral damage.

## Conclusions

PFA is a promising option for treating non-PV foci, particularly those originating from the inferior-posterior left atrial wall adjacent to the esophagus. It reduces the risk of esophageal injury while creating effective and broad lesions.
